# Relative Bioavailability of Cadmium in Rice: Assessment, Modeling, and Application for Risk Assessment

**DOI:** 10.3390/foods12050984

**Published:** 2023-02-26

**Authors:** Likun Yang, Xiaoyue Zhang, Di Zhao, Peng Wang, Fangjie Zhao

**Affiliations:** College of Resources and Environmental Sciences, Nanjing Agricultural University, Nanjing 210095, China

**Keywords:** cadmium, rice, relative bioavailability, risk assessment, food safety

## Abstract

Rice consumption is the primary route of cadmium (Cd) exposure to the populations with rice as the staple food. To accurately assess the potential health risks of Cd exposure via rice consumption, determination of Cd relative bioavailability (RBA) in rice is necessary. However, large variations exist in Cd-RBA, hindering the application of source-specific Cd-RBA values to different rice samples. In this study, we collected 14 rice samples from Cd contaminated areas and determined both rice compositions and Cd-RBA using in vivo mouse bioassay. Total Cd concentration varied from 0.19 to 2.54 mg/kg in the 14 rice samples, while Cd-RBA in rice ranged from 42.10% to 76.29%. Cadmium-RBA in rice correlated positively with calcium (Ca) (R = 0.76) and amylose content (R = 0.75) but negatively with the concentrations of sulfur (R = −0.85), phosphorus (R = −0.73), phytic acid (R = −0.68), and crude protein (R = −0.53). Cd-RBA in rice can be predicted by Ca and phytic acid concentrations in a regression model (R^2^ = 0.80). Based on the total and bioavailable Cd concentrations in rice, weekly dietary Cd intake for adults was estimated to be 4.84–64.88 and 2.04–42.29 μg/kg bw/week, respectively. This work demonstrates the possibility of Cd-RBA prediction based on rice compositions and provides valuable suggestions for health risk assessment with consideration of Cd-RBA.

## 1. Introduction

Cadmium (Cd) is a toxic element present in the environment from both geogenic and anthropogenic sources [1,2]. Among multiple exposure pathways, dietary intake is the primary route of Cd exposure for the general non-smoking population, accounting for ~90% of the total Cd exposure [3]. Chronic Cd exposure via oral ingestion has been linked to increased risk of hypertension, osteoporosis, kidney damage, and even cancers [4,5], raising concerns about the potential adverse health effects of Cd exposure via food consumption.

Among different food categories, rice is the largest contributor to the dietary Cd intake in China [6]. In some polluted areas, the average Cd concentration in rice was up to 0.69 mg/kg (range: 0.005–4.80 mg/kg), with 56–87% of the rice samples exceeding the Chinese food limit of 0.20 mg/kg [7,8]. These Cd concentrations in rice are similar or even higher than those 2,446 rice samples (mean Cd: 0.38 mg/kg) collected from the Jinzu River basin in Toyama prefecture, Japan, where the “itai-itai” disease (kidney failure and softening of bones) occurred. “Itai-itai” disease is the most severe stage of chronic Cd poisoning in humans caused by prolonged oral ingestion of rice, which was contaminated with Cd from irrigation waters polluted by mining activities upstream [9,10]. These surveys highlight the serious pollution status of Cd in some polluted areas and the urgent need to evaluate the potential health risks of Cd exposure for local residents. 

Traditional risk assessment typically considers only total Cd concentration in rice, resulting in overestimation of the potential health risks. Recently, relative bioavailability (RBA) of Cd (the percentage of Cd in rice that is absorbed into the systemic circulation after oral ingestion) has been incorporated into risk assessment to improve the accuracy of the risk assessment [11,12,13]. For example, a one-compartment toxicokinetic model has been frequently used to predict urinary Cd level based on dietary Cd intakes [14,15]. When total Cd concentration in rice was used for 119 non-smokers from a Cd-contaminated area who consume rice as the staple food, their predicted urinary Cd (geometric mean: 4.14 μg/g creatinine) was 3.5-fold higher than the measured urinary Cd (geometric mean: 1.20 μg/g creatinine) [13]. After incorporating Cd-RBA in rice (17–57%), the predicted urinary Cd was close to the measured value (1.07 vs. 1.20 μg/g creatinine), highlighting the importance of bioavailability in accurately estimating human Cd exposure and related health risks. However, large variations in Cd-RBA among different rice samples have been reported [13,16,17], hindering the application of determined Cd-RBA values in rice from specific sources to different rice samples. 

Due to the expensive cost and ethical considerations of in vivo animal bioassays, simple, rapid, and inexpensive in vitro methods have been developed as a substitute for in vivo bioassays once they were validated using in vivo animal studies [11,18]. However, for Cd in rice, correlations between determined Cd-RBA using in vivo mouse bioassays and determined Cd bioaccessibility using four in vitro methods were poor (R^2^ = 0.0006 − 0.52), indicating that the current in vitro methods may be unreliable to predict Cd-RBA for rice [17]. Therefore, in addition to in vitro assays, an alternative approach is to develop reliable predictive models based on rice compositions as a rapid screening tool for the assessment of Cd-RBA in rice.

In this study, a total of 14 rice samples were collected from Cd-contaminated areas in southern China. The specific objectives of this study were to (1) determine Cd-RBA in rice samples from Cd-contaminated areas; (2) develop a prediction model of rice Cd-RBA using rice compositions as predictors; (3) and assess the potential health risks of Cd exposure for local populations via rice consumption.

## 2. Materials and Methods

### 2.1. Collection and Preparation of Rice Samples

Rice samples were collected from local farmers living in two villages contaminated with Cd due to nearby mining activities or the prolonged irrigation of Cd-contaminated water in Hunan Province, China. After excluding those rice samples insufficient for in vivo mouse bioassays and those purchased from supermarkets and farm markets, only 14 locally grown rice samples were included in this study. The Cd concentrations in the rice samples (0.19 to 2.54 mg/kg) included in this study are similar to those previously reported, which is typical of the region [7,8]. Once the 14 whole grains were transferred to the lab, they were dehusked and then polished using a laboratory rice milling machine. For each rice sample, ~120 g of polished rice was washed thoroughly with Milli-Q water three times. Following washing, the rice was cooked with 240 mL of Milli-Q water using an intelligent germinated rice machine for ~25 min. Cooked rice samples were then removed from the cooker, stored at −80 °C, and freeze-dried. All the rice samples were ground to a fine powder and mixed thoroughly. 

### 2.2. Chemical Analysis of Rice Samples 

Briefly, 0.20 g cooked rice powder were digested with 5 mL of high purity HNO_3_ in a microwave digestion system [19,20]. The concentrations of Cd and other elements, including calcium (Ca), iron (Fe), zinc (Zn), sulfur (S), and phosphorus (P), in the digests were determined based on inductively coupled plasma mass spectrometry (ICP-MS, Perkin Elmer NexION 300X, USA). Quality assurance and control were conducted during metal analysis. Briefly, indium isotope (^114^In) was used as an internal standard, which was added to the blanks, calibration standards, and samples to compensate for the long-term signal drift of the instrument and the matrix effects. The accuracy of the ICP-MS method used for Cd analysis was validated using a standard reference material for rice (GBW10045), with a measured Cd concentration of 0.31 ± 0.02 mg/kg in rice GSB-23 compared to the standard concentration of 0.32 ± 0.04 mg/kg. Furthermore, spikes and duplicates were included in every 20 samples during analysis using ICP-MS, with recoveries being 92–103% and 93–105%, respectively. 

The content of phytic acid in rice was determined based on the method of GB 5009.153-2016 [21]. In brief, phytic acid was extracted by shaking 10 g of rice flour in 40 mL sodium sulfate–hydrochloric acid (0.10 g/mL) for 2 h. The extract was centrifuged at 500 r/min for 5 min, and the supernatant was collected and made up to 50 mL using sodium sulfate–hydrochloric acid. The filtered solution was then eluted and purified by anion exchange resin. The phytic acid concentration in the eluate was determined by a colorimetric method after reaction with the ferric trichloride–sulfosalicylic acid mixture. The absorbance at a wavelength of 500 nm was measured using a spectrometer (Shimadzu UV-1800), and the content of phytic acid was quantified against standard curves of sodium phytate. Rice powders were analyzed with a near-infrared spectrometer (Perten DA7250) to measure the content of crude protein and amylose [22]. 

### 2.3. Relative Bioavailability of Cd in Rice 

A steady state dosing method with free access to diet was used for in vivo bioavailability study. Briefly, the 14 samples of cooked rice powder were thoroughly mixed with Milli-Q water and made into a paste by kneading. Then, the amended mouse feed was evenly divided into small pieces before being freeze-dried. In addition, CdCl_2_ was added into a non-contaminated rice (Cd: 0.002 mg/kg) using a similar method to produce different Cd concentrations (0.20–5.00 mg/kg) to assess the dose responses. To ensure that the amended mouse feed was thoroughly mixed, Cd concentrations in randomly selected pellets for each rice sample were determined, resulting in a relative standard deviation < 5%.

A total of sixty female Balb/c mice with body weights of 18–20 g were purchased. Following acclimation for 1 week under a standard condition (25 °C, 50% humidity and 12:12 h light/dark cycle) with free access to Milli-Q water and mouse feed. Milli-Q water was used to avoid metal intakes from drinking water. For the basal mouse feed used during acclimation, Cd concentration was 0.002 ± 0.002 mg/kg. This background level of Cd is only 0.78–1.05% of those in the rice samples tested. Mice were fasted overnight, weighed, and randomly assigned to individual plastic cages. Animal care was compliant with the guide for the care and use of laboratory animals and approved by the Ethics Committee of Animal Experiments of Nanjing Agriculture University. 

For Cd exposure, each mouse received ∼3 g of amended feed at 9:00 am every day. For each rice sample and CdCl_2_ dose levels, 3 mice were used as replicates. During the 10-days exposure, mice had free access to water and amended feed ad libitum. At the end of a 10-day exposure, remaining rice pellets were removed from cages, freeze-dried, and weighed to calculate the food consumption rate. Mice were fasted overnight and sacrificed to collect the liver and kidneys. The liver and kidneys were stored at −80 °C, freeze-dried, digested, and then measured for Cd concentration using ICP-MS. Non-contaminated rice was fed to mice to determine background values of Cd accumulation in mouse liver and kidneys. Cd-RBA in rice was calculated using Equation (1)

(1) $$\mathrm{Cd}\;\mathrm{relative}\;\mathrm{bioavailability}\;\left(\%\right)=\left(\frac{\mathrm{Tissue}\;{\mathrm{Cd}}_{\mathrm{rice}}}{{\mathrm{Cd}\;\mathrm{dose}}_{\mathrm{rice}}}\times \frac{{\mathrm{Cd}\;\mathrm{dose}}_{\mathrm{CdCl}2}}{\mathrm{Tissue}\;{\mathrm{Cd}}_{\mathrm{CdCl}2}}\right)\;\times 100\%$$

where Tissue Cd_rice_ and Tissue Cd_CdCl2_ are Cd concentrations accumulated in mouse liver plus kidneys following the consumption of rice and CdCl_2_-spiked rice; Cd dose_rice_ and Cd dose_CdCl2_ are Cd dose levels from rice and CdCl_2_-spiked rice exposure [13,23]. 

### 2.4. Prediction Model of Cd-RBA in Rice

The prediction model of Cd-RBA in rice was derived using rice compositions as predictors, including concentrations of Ca, Fe, Zn, S, P, Cd, amylose, crude protein, and phytic acid. Stepwise multiple linear regression analysis was conducted in R software (version 3.4.2) to determine the regression parameters, based on 14 pairs of data of Cd-RBA and rice compositions. The best-fitted regression model is presented in Equation (2):

(2) $$\mathrm{lg}\left({\mathrm{Cd}}_{\mathrm{RBA}}\right)=a+b\times \mathrm{lg}\left(\mathrm{Ca}\right)+c\times \mathrm{lg}\left(\mathrm{phytic}\;\mathrm{acid}\right)$$

where Cd_RBA_ is the relative bioavailability of Cd in rice (%), Ca is expressed in mg/kg, and phytic acid is expressed in g/kg, respectively. 

### 2.5. Calculation of Weekly Dietary Cd Intake 

To assess the potential health risks of Cd exposure via rice consumption, weekly dietary Cd intake was calculated from total or bioavailable Cd in rice following Equations (3) and (4).

(3) $$\mathrm{Weekly}\;\mathrm{dietary}\;\mathrm{Cd}\;\mathrm{intake}=\frac{C\times \mathrm{IR}}{\mathrm{BW}}$$

(4) $$\mathrm{Adjusted}\;\mathrm{weekly}\;\mathrm{dietary}\;\mathrm{Cd}\;\mathrm{intake}=\frac{C\times \mathrm{IR}\times \mathrm{RBA}}{\mathrm{BW}}$$

where C is the total Cd concentration in rice (mg/kg), IR is the consumption rate of rice (g/week), BW is the body weight of consumers (kg), and RBA is the relative bioavailability of Cd in rice (%). The rice consumption rate of 218.60 g/d for the general population was obtained from the Chinese national nutrition and health survey, the average body weight of 60 kg for adults was used for calculation [24]. The calculated weekly dietary Cd intake was compared to the provisional tolerable weekly intake for Cd of 5.80 μg/kg bw/week proposed by the Joint FAO/WHO Expert Committee on Food Additives (JECFA). 

### 2.6. Statistical Analysis

All concentrations in rice samples and rice Cd-RBA are presented as means and standard deviations (SD) of the three replicates. A significant difference in rice Cd concentrations and Cd-RBA between rice samples was assessed using Fisher’s least-significant difference (LSD) with a significance level of *p* < 0.05. All statistical statistics were performed using IBM SPSS Statistics, version 28. All graphs were performed using SigmaPlot (version 14.0) and Origin 2023. 

## 3. Results

### 3.1. Chemical Compositions in Rice

**Figure 1:**
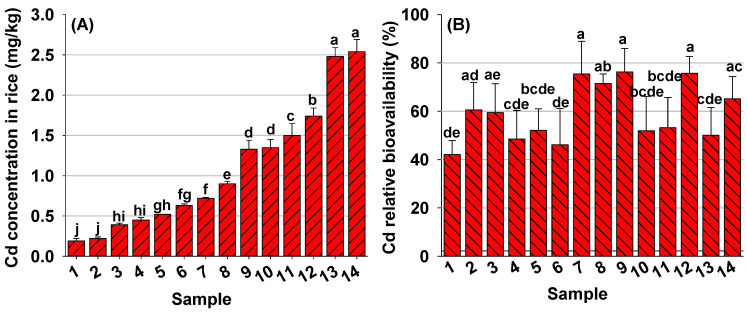
Cadmium concentration (**A**) and Cd relative bioavailability (**B**) in 14 rice samples. Different letters above bars indicate significant (*p* < 0.05) difference among rice samples.

In this study, Cd concentrations in the 14 polished rice samples collected from Cd contaminated areas varied from 0.19 to 2.54 mg/kg (mean = 1.07 mg/kg) (Figure 1A; Table S1), with 93% of the samples exceeding the limit of 0.20 mg/kg. 

Besides Cd, the concentrations of chemical elements and compositions, such as Ca, Fe, Zn, S, P, crude protein, amylose, and phytic acid, in the rice samples were also measured (Table 1). The average concentrations of Ca, Fe, and Zn were 120.16 mg/kg, 5.07 mg/kg, and 14.33 mg/kg, respectively. The average concentrations of S and P were 1499 mg/kg and 1393 mg/kg, respectively. The average contents of crude protein and amylose were 10.66% and 26.32%, respectively. The average concentration of phytic acid was 1.59 g/kg, ranging from 0.91 to 2.69 g/kg.

### 3.2. Relative Bioavailability of Cd in Rice 

To accurately assess the potential health risks of Cd exposure via rice consumption, Cd-RBA in the 14 rice samples was assessed using a steady state in vivo mouse bioassay. A dose-response relationship was first established between Cd doses and Cd accumulations in mouse liver and kidneys. Following the administration of different concentrations of CdCl_2_ (0.20–5.00 mg/kg) to mice over a 10-day exposure, a strong linear relationship (R^2^ = 0.97) between the Cd dose and the concentration of Cd accumulated in mouse liver plus kidneys was observed (Figure S1), confirming the suitability of the combined end point (liver plus kidneys) to determine Cd-RBA in rice at similar Cd doses. For the 14 rice samples, Cd-RBA varied from 42.10% to 76.29%, with the average being 59.16% (Figure 1B). 

**Figure 2:**
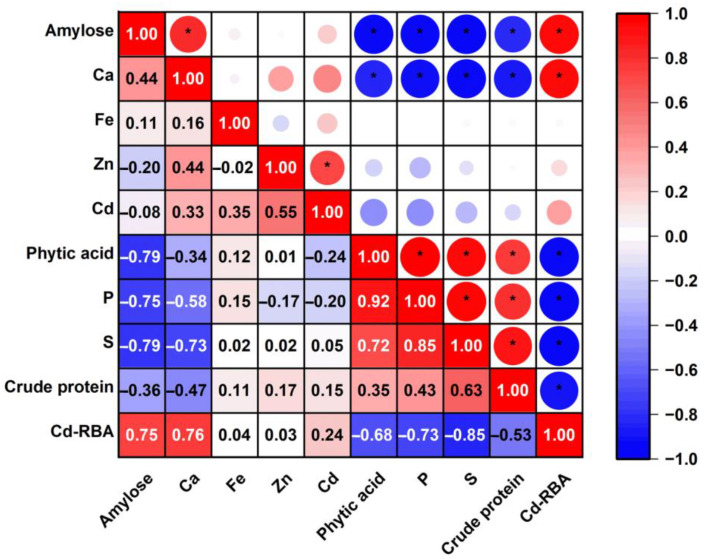
Pearson rank correlation between Cd-RBA and chemical composition in rice. Significant correlations are denoted by “*” (*p* < 0.05).

In addition, the correlations between rice Cd-RBA and rice compositions were analyzed (Figure 2). For mineral nutrient elements in rice, Cd-RBA was significantly correlated with Ca concentrations (R = 0.76) but not with Fe or Zn concentrations (R = 0.04 and 0.03). Cadmium-RBA was strongly and negatively correlated with the concentrations of S (R = −0.85) and P (R = −0.73). No significant correlation (R = 0.24) was observed between Cd-RBA and Cd concentrations in the rice samples. For rice compositions in rice samples, Cd-RBA correlated positively with the percentage of amylose (R = 0.75) but negatively with phytic acid (R = −0.68) and crude protein (R = −0.53). 

### 3.3. Using Rice Compositions to Predict Cd-RBA in Rice 

Multiple linear regression analysis was then performed between Cd-RBA and rice compositions. Rice Ca and phytic acid were found to be the two major factors influencing Cd-RBA in rice, as shown in the following Equation (5):

(5) $$\mathrm{lg}\left({\mathrm{Cd}}_{\mathrm{RBA}}\right)=0.9+0.44\times \mathrm{lg}\left(\mathrm{Ca}\right)-0.3\times \mathrm{lg}\left(\mathrm{phytic}\;\mathrm{acid}\right)\;\left(R^{2}=0.80,\;p<0.05\right)$$

**Figure 3:**
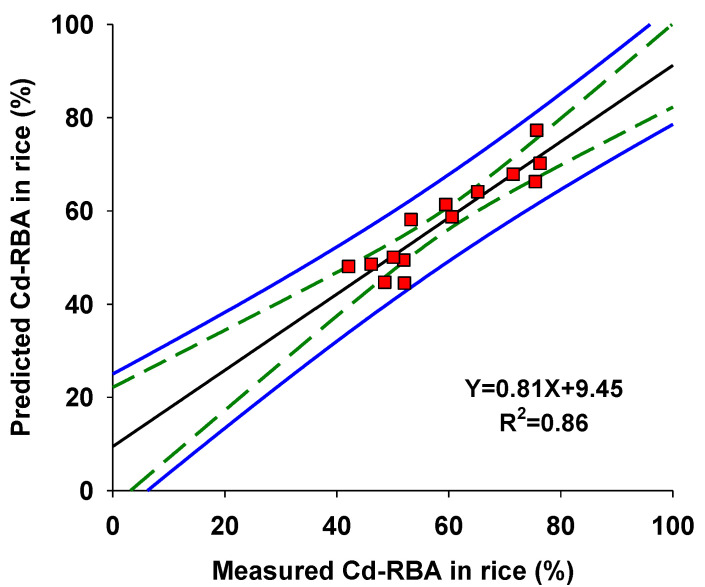
Correlations between measured and predicted Cd-RBA based on Ca and phytic acid concentrations in rice. Blue solid lines and green dashed lines represent 95% prediction and confidence bands, respectively.

The data highlighted a significant correlation between rice Cd-RBA and the concentrations of Ca and phytic acid, accounting for 80% of the variability in Cd-RBA for all tested rice samples. In the regression equation obtained, the influence of Ca on Cd-RBA was positive, whereas the influence of phytic acid was negative. Based on the multiple linear regression, the predicted Cd-RBA values were obtained and compared with measured Cd-RBA using linear regression. A strong significant relationship (R^2^ = 0.86) between predicted Cd-RBA and measured Cd-RBA was obtained (Figure 3), providing a reliable predictor of Cd-RBA based on rice compositions.

### 3.4. Health Risk Assessment Based on Total Cd and Bioavailable Cd in Rice

**Figure 4:**
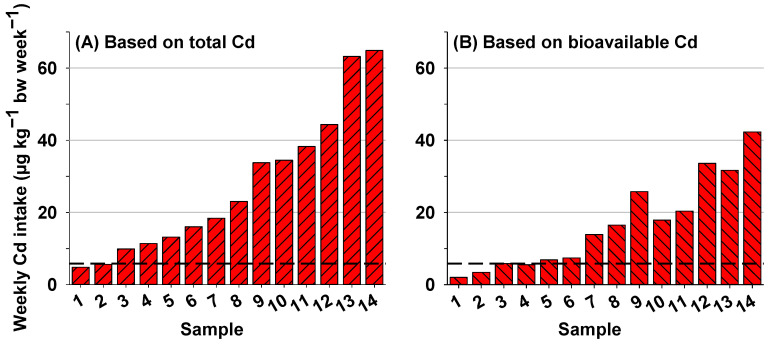
Weekly dietary Cd intake levels estimated using total Cd concentration in rice (**A**) and bioavailable Cd concentration in rice (**B**). Weekly dietary Cd intake via oral ingestion of rice was calculated for an adult with a body weight of 60 kg and a rice consumption rate of 1.53 kg/week. The black dashed lines indicate the tolerable intake level of 5.80 μg/kg bw/week for Cd proposed by JECFA to protect human health.

To assess the potential health risk of Cd exposure via rice consumption, weekly dietary Cd intake for an adult with a body weight of 60 kg was estimated based on total Cd and bioavailable Cd concentrations assuming the rice consumption rate of 218.6 g/day (Figure 4) [24]. Based on total Cd, consumption of rice would result in a weekly Cd intake of 4.84–64.88 μg/kg bw/week (Figure 4A). The estimated weekly dietary Cd intake from 12 of 14 rice samples exceeded the provisional tolerable weekly intake level of 5.80 μg/kg bw/week for Cd intake proposed by JECFA to protect humans from adverse health effects. When taking bioavailable Cd into consideration, the estimated weekly dietary Cd intake was reduced to 2.04–42.29 μg/kg bw/week (Figure 4B), which is 23.71–57.90% lower compared to total Cd.

## 4. Discussion

The 14 rice samples used in the present study contained elevated levels of Cd due to soil contamination from nearby mining activities or the prolonged irrigation of Cd-contaminated water. The Cd concentrations in the rice samples (0.19 to 2.54 mg/kg) are typical of the region [7,8] and likely lead to elevated dietary Cd intakes and health risk for the local residents. Potential health risk of Cd exposure depends not only on the total amount of Cd intake but also on the bioavailability of Cd in the ingested food. The large variation in Cd-RBA (42.10–76.29%) among the 14 rice samples detected in this study is similar or higher than those of rice Cd-RBA reported by previous studies. For example, Zhao et al. [13] first determined Cd-RBA in rice using an in vivo mouse bioassay and found large variability (17–57%) in Cd-RBA among 10 rice samples (Cd: 0.29–1.09 mg/kg) collected from Cd-contaminated areas caused by enamel pottery production. Higher Cd-RBA values of 41–84% have been reported for 11 rice samples (Cd: 0.41–1.67 mg/kg) collected from mining/smelting areas based on in vivo mouse bioassay [25], which is similar to the values detected in this study. Zhuang et al. [17] collected six rice samples from supermarkets, farmer markets near mining-impacted areas, and greenhouses, which varied widely in Cd concentration (0.15–10.10 mg/kg). An in vivo mouse bioassay was conducted using these rice samples, with Cd-RBA being 15–56%, 18–56%, and 3.71–54% when kidneys, liver, and femur were used as the end point. A recent study showed large variations (36–97%) in Cd-RBA among three rice samples with relatively low Cd concentrations (0.10–0.40 mg/kg) when different end points (kidneys, liver, and liver plus kidneys) were used [16]. These studies together showed a large variation in Cd-RBA between rice samples (3.7–97.3%), hindering the accurate health risk assessment of Cd exposure via rice consumption when incorporating of Cd-RBA. However, the main factors affecting Cd-RBA in rice is unclear, it is imperative to identify key factors influencing Cd-RBA in rice.

The large variation in Cd-RBA among different rice samples may be related to differences in rice mineral element concentrations because Cd utilizes the same intestinal transporters as mineral elements, including Zn, Fe, and Ca [26,27]. Higher concentrations of Ca, Fe, or Zn in rice may decrease Cd-RBA due to competitions for transporters in the intestinal epithelium [28,29]. However, previous studies showed no strong correlations between Cd-RBA and nutrients elements, such as Ca, Fe, and Zn concentrations in rice [17,25]. In contrast, we found a positive correlation between Cd-RBA and Ca concentration. The intestinal transporters for Ca absorption, such as Ca binding protein (CaBP), have been reported to be able to absorb Cd since the binding affinities of CaBP for Cd and Ca are similar [30]. Rats fed on low Ca diets were found to have a ∼60% higher Cd accumulation in the kidneys and liver than those fed on a normal diet of Ca intake, likely because synthesis of CaBP is intensified at low dietary Ca intake [31]. However, contrary to the expected negative effect of Ca on Cd absorption, we found Cd-RBA in rice increased with Ca concentration. The potential mechanism underpinning the contrary effect of Ca on Cd-RBA in rice is unknown, which warrants further investigation.

Besides mineral nutrient elements, the large variation in rice Cd-RBA may be accounted by differences in S and P concentrations in rice. Using synchrotron X-ray absorption spectrometry, Gu et al. [20] showed that the majority (66–92%) of Cd in rice was combined with thiol-rich proteins, presenting as Cd-thiolate complexes. The negative correlation between Cd-RBA and rice S concentrations suggests that complexation of Cd by thiol-containing compounds in rice may reduce the bioavailability of Cd. Besides the effect of S, an in vivo mouse experiment showed that Cd accumulation in kidneys was markedly reduced by increasing the P concentration [32]. The formation of cadmium phosphate precipitates could explain the decreased Cd-RBA in rice with an increasing P concentration, although further research is needed to elucidate the potential mechanism.

Rice compositions, such as the content of amylose, phytic acid, and crude protein, may also contribute to differences in Cd-RBA in rice. Rice contains high levels of starch; the main difference in starch composition among different rice varieties is related to the variation in the proportions of its two polymers, amylopectin and amylose [33]. There is a high possibility that higher Cd-RBA with increasing amylose is caused by the interaction with other components since amylose content was found to correlate with Ca, S, P, phytic acid, and crude protein content (Figure 2). It has been demonstrated that phytic acid, which is a strong chelator of divalent cations, inhibits the intestinal absorption of metal cations, such as Ca, Fe, and Zn [34,35]. Lee et al. [34] found that the content of phytic acid in rice was negatively correlated with mineral bioaccessibility, with the correlation being the highest in Ca (R = 0.60), followed by Fe (R = 0.40), and Zn (R = 0.27). The Ca-phytate complex has a strong affinity for Cd, which could lead to reduced Cd-RBA in rice. An in vitro intestinal study showed a significant reduction in Cd absorption in the presence of phytic acid [35], which is consistent with our study. Protein content is another factor that may affect Cd bioavailability. In a mouse bioassay using renal accumulation of Cd as the endpoint [32], cadmium concentration in the kidneys was significantly decreased by high protein compared to low protein diet (25% vs. 10%). 

Thus, rice compositions were used as predictors for Cd-RBA based on multiple linear regression analysis; Ca and phytic acid were selected as the more important parameters in the prediction model. The prediction model was validated by comparing the measured and predicted Cd-RBA in 14 rice samples. All the measured values of Cd-RBA in rice fell within the 95% prediction interval. The developed model was therefore regarded as a reliable model for predicting Cd-RBA in rice collected from other sources once the rice compositions were determined. 

Following determination of Cd-RBA in rice, weekly dietary Cd intake via rice consumption was estimated using total and bioavailable Cd concentrations to assess the potential health risk of Cd exposure. When total Cd in rice was used for calculation, weekly dietary Cd intake increased with an increasing rice Cd concentration, indicating increasing potential health risk with an elevated rice Cd concentration. Unlike the linear relationship between rice total Cd concentration and weekly dietary Cd intake, a higher Cd concentration in rice samples may not always lead to a higher health risk when Cd-RBA is taken into account. For example, rice #13 had a higher Cd concentration than rice #12 (2.48 vs. 1.74 mg/kg) but would lead to a lower Cd intake level when Cd-RBA is considered (31.68 vs. 33.60 μg/kg bw/week). Similar findings have been reported for other food matrices, such as leafy vegetables [12], highlighting the importance of incorporating Cd-RBA into the health risk assessment. Thus, Cd-RBA in rice is recommended in future risk assessment to improve the accuracy and reliability.

## 5. Conclusions

A large variation in Cd-RBA was observed among 14 rice samples based on in vivo mouse bioassay. Cd-RBA positively correlated with Ca and amylose content in rice, while negatively correlated with concentrations of S, P, phytic acid, and crude protein. Multiple linear regression analyses showed that Ca and phytic acid concentrations are the two major factors influencing Cd-RBA, demonstrating the possibility of using rice compositions to predict Cd-RBA in rice. Further studies are required to elucidate the mechanisms underpinning the effect of Ca, amylose, S, P, phytic acid, and crude protein on Cd-RBA. As Cd-RBA varies widely, rice samples with high Cd concentrations do not always mean high health risks of Cd exposure. To improve the accuracy of a health risk assessment, incorporation of bioavailability information is strongly suggested.

## Figures and Tables

**Table 1:** Chemical composition analysis of 14 rice samples.

Compositions	Mean ± SD	Range
Cd (mg/kg)	1.07 ± 0.78	0.19–2.54
Ca (mg/kg)	120.16 ± 31.20	79.49–165.71
Fe (mg/kg)	5.07 ± 1.32	2.60–7.37
Zn (mg/kg)	14.33 ± 2.88	11.35–21.22
S (mg/kg)	1499.72 ± 197.52	1183.81–1813.53
P (mg/kg)	1393.48 ± 434.01	878.93–2345.57
Crude protein (%)	10.66 ± 0.70	9.31–12.36
Amylose (%)	26.32 ± 4.62	18.55–32.64
Phytic acid (g/kg)	1.59 ± 0.56	0.91–2.69

## Data Availability

The data presented in this article are available from the corresponding authors upon reasonable request.

## Supplementary Materials

The following supporting information can be downloaded at: https://www.mdpi.com/article/10.3390/foods12050984/s1, Figure S1: Linear dose response of Cd accumulation in liver plus kidneys to Cd dose levels following 10-d consumption of CdCl_2_-amended diets at 0.2–5.0 mg/kg Cd. Table S1: Concentrations of Cd and nutrient elements in the 14 rice samples tested for in vivo mouse bioassay.
