# Supplementary material for: Relative Bioavailability of Cadmium in Rice: Assessment, Modeling, and Application for Risk Assessment
Source: Foods. 2023 Feb 26;12(5):984. doi: 10.3390/foods12050984 (PMC10000470; doi:10.3390/foods12050984)
Supplement: Supplementary file 1 [file foods-12-00984-s001.zip › foods-2196753-supplementary.pdf]

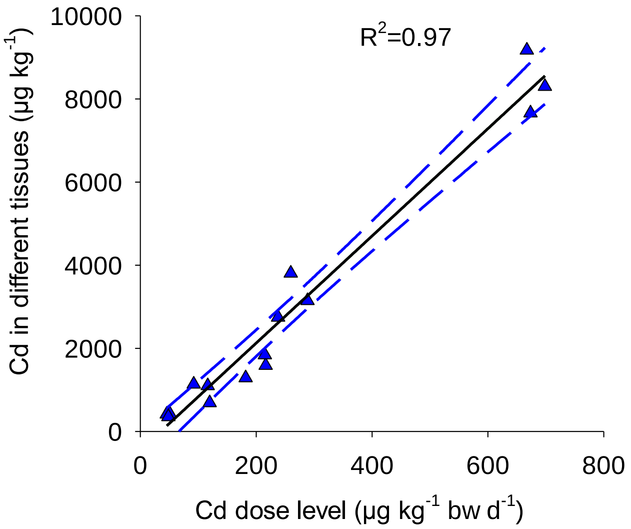

Figure S1. Linear dose response of Cd accumulation in liver plus kidneys to Cd dose levels following 10-d consumption of $CdCl_2$-amended diets at 0.2–5.0 mg/kg Cd.

Table S1. Concentrations of Cd and nutrient elements (mean ± SD) in the 14 rice samples tested for in vivo mouse bioassay.

Rice Sample	Cd (mg/kg)	Ca (mg/kg)	Fe (mg/kg)	Zn (mg/kg)
#1	0.19 ± 0.03	90.49 ± 18.44	5.32 ± 1.23	15.54 ± 0.07
#2	0.22 ± 0.02	105.88 ± 8.84	5.54 ± 0.23	13.67 ± 0.83
#3	0.39 ± 0.02	135.31 ± 5.58	4.92 ± 1.55	11.87 ± 0.21
#4	0.45 ± 0.03	99.78 ± 11.61	5.40 ± 0.19	11.70 ± 0.19
#5	0.52 ± 0.003	94.60 ± 16.24	6.08 ± 0.59	11.35 ± 0.45
#6	0.63 ± 0.02	79.49 ± 5.81	2.61 ± 0.45	12.57 ± 0.63
#7	0.72 ± 0.01	131.68 ± 4.48	2.85 ± 0.30	13.18 ± 0.18
#8	0.90 ± 0.03	140.87 ± 8.94	3.44 ± 1.05	15.04 ± 0.94
#9	1.33 ± 0.11	153.31 ± 4.37	5.46 ± 3.24	14.84 ± 0.66
#10	1.35 ± 0.10	91.53 ± 14.97	5.79 ± 3.00	11.55 ± 0.96
#11	1.50 ± 0.15	162.02 ± 7.56	4.56 ± 1.02	21.22 ± 1.66
#12	1.74 ± 0.10	165.71 ± 15.65	7.37 ± 2.61	12.65 ± 0.50
#13	2.48 ± 0.11	82.13 ± 6.91	5.45 ± 0.61	16.12 ± 1.12
#14	2.54 ± 0.15	149.51 ± 4.94	6.17 ± 0.11	19.34 ± 0.51
